# Early and mid-term outcomes of open thoracoabdominal aortic aneurysm repair after thoracic endovascular aortic repair

**DOI:** 10.1186/s12872-024-03837-8

**Published:** 2024-03-26

**Authors:** Ji Lin, Wei Liu, Cheng-Wei Yang, Kaitao Jian, Yu Xia, Hao Peng, Bin You, Li-Zhong Sun

**Affiliations:** 1 Department of Cardiovascular Surgery, Beijing Anzhen Hospital, Capital Medical University, Beijing Institute of Heart Lung and Blood Vessel Diseases, Beijing, China; 2 Department of Cardiovascular Surgery, DeltaHealth Hospital Shanghai, Shanghai, China

**Keywords:** Thoracoabdominal aortic aneurysm, Endovascular repair, TEVAR, Secondary open surgery

## Abstract

**Objective:**

To evaluate the early and mid-term outcomes of open repair in patients with thoracoabdominal aortic aneurysm (TAAA) after thoracic endovascular aortic repair (TEVAR).

**Methods:**

This was a retrospective single center study. Data were retrospectively collected and analyzed for consecutive patients undergoing open TAAA repair (TAAAR) after TEVAR from November 2016 to June 2021. Indications for TAAAR included aneurysm progression due to endoleak, persisted false lumen perfusion, proximal/distal disease progression, and aorta rupture. The risk factor of operative mortality was analyzed by multivariable logistic regression model and the survival was evaluated by Kaplan–Meier.

**Results:**

Sixty-three patients who met the inclusion criteria for the study were identified. The mean age at TAAAR was 41 ± 12 years and 43 (68.3%) were male. Marfan syndrome (MFS) was presented in 39 patients (61.9%). 60 (95.2%) patients presented with post-dissection aneurysm and 3 (4.8%) patients with degenerative aneurysm. The extent of TAAA was Crawford I in 9 (14.3%), II in 22 (34.9%), III in 23 (36.5%), and IV in 9 (14.3%). Emergent TAAAR was done in 10 (15.9%) patients, and deep hypothermic circulatory arrest was used in 22 (34.6%). Endograft was explanted in 31 (49.2%). Operative mortality was 11 (17.5%). Stroke, paraplegia, and acute kidney failure occurred in 5 (7.9%), 7 (11.1%), and 6 (9.5%) patients, respectively. Pulmonary complications occurred in 19 (30.2%) patients. The estimated survival was 74.8 ± 4.9% at 5 years. Late reoperations were performed in 2 patients at 2.5 years and 1.3 years, respectively.

**Conclusions:**

In this series of TAAA after TEVAR, TAAAR was related with a high risk of operative mortality and morbidity and the midterm outcomes represented a durable treatment and were respectable.

**Supplementary Information:**

The online version contains supplementary material available at 10.1186/s12872-024-03837-8.

## Introduction

Since the first case of thoracic endovascular aortic repair (TEVAR) for the treatment of acute aortic dissection was successfully performed in 1996 [1], the procedure has been gaining in popularity due to its superiority of low invasiveness and favorable short-term results. TEVAR has been widely applied in thoracic aortic diseases [2–5]. Although complications and failures can occur after TEVAR, they can again be fixed by endoluminal approaches because of the ongoing improvement of endovascular technique [6, 7]. However, when further endovascular repairs are unsuitable to address these complications and failures, a relevant number of patients remain who require conventional open repair to fix it.

Due to additional technical difficulties faced during surgery, the high morbidity and mortality were relevant to open thoracic and thoracoabdominal aortic aneurysm repair (TAAAR) after TEVAR [8, 9]. Additionally, literature focusing on the safety and durability of open TAAAR after primary TEVAR is scarce [8].

This study presents the early and follow-up outcomes of open TAAAR after TEVAR as a secondary treatment and highlights clinical experiences to contribute to the field.

## Patients and methods

### Patients

This study was approved by the Ethics Committee of Beijing Anzhen Hospital, Capital Medical University, and Shanghai Delta Hospital. (No. 2019030X). This work was reported in accordance with the STROBE criteria [10]. Data were retrospectively collected for consecutive patients who underwent open TAAAR after previous TEVAR through a search of our medical records from November 2016 to June 2021 in Shanghai DeltaHealth Hospital which is a high-volume tertiary referral center for the treatment of complex aortic diseases in China. All informed consent for patients was waived. Indications for TAAAR included aneurysm progression (size > 50 mm) due to endoleak, persisted false lumen perfusion, proximal/distal disease progression, and aorta rupture. The reasons for open repairs instead of endovascular approach were as following: 1. Patients with connective tissue disease, such as Marfan syndrome; 2. The landing zone for the stent was not enough; 3. Patients with extensive TAAA (type II and type III) involving celiac artery branches; 4. A multidisciplinary discussion concluded that the patient was not suitable for endovascular treatment or that endovascular therapy had poor long-term outcomes. Patients with endovascular graft infectious or with a fistula between the esophagus and thoracic aorta were excluded from this study. The extent of the aortic repair was graded according to the original Crawford classification [11].

### Surgical techniques

All patients were posed in the right lateral position and separate bronchial ventilation was applied for collapsing the left lung during the procedure. Operations were performed through a left thoracoabdominal incision. The level of the thoracic incision was based on the proximal extent of the surgery. Deep hypothermic circulatory arrest (DHCA) was used when cross-clamping was difficult for controlling the proximal descending aorta. The procedure was induced at a nasopharyngeal temperature of 18 °C and without cerebral perfusion and cardioplegia fluid. All patients underwent prosthetic replacement of the diseased aorta with a four-branch graft.

Heparin was given at 3 mg/kg with an ACT greater than 480 s in the procedure with femoro-femoral bypass. In the case of aorto-iliac bypass or simple aortic clamping, Heparin was given at 1 mg/kg with an ACT greater than 200 s. The distal artery and visceral perfusion strategy included sequential aortic clamping combined with aorto-iliac bypass [12] or femoro-femoral bypass [11] (simple aortic clamping was only used in parts of less extensive TAAAR). In brief, femoro-femoral bypass was applied with cardiopulmonary bypass and cannulation in the femoral artery and vein. The main procedure of the aorto-iliac bypass was as follows. First, two branches of the our-branch graft were sequentially sutured to the bilateral common iliac arteries in an end-to-side fashion. Next, the proximal aorta was cross-clamped and sutured to the trunk of the graft in an end-to-end fashion. This technique permits distal perfusion through the aorto-iliac bypass while performing subsequential clamping. Before anastomosis of the visceral branches, the aorta was clamped above the diaphragm while the perfusion of the renal artery and the abdominal artery was provided by the femoro-femoral bypass or aorto-iliac bypass.

The preservation of the proximal descending aortic segment of stent grafts mainly depends on the safety and feasibility of the anastomotic zone and good remodeling between the endograft and descending aorta. In general, if the procedure involves a region of visceral branches, we usually reattached the visceral branches in the order of the superior mesenteric artery (SMA), right renal artery (RA), left RA, and celiac artery (CA).

Protective adjuncts were used for safeguarding the spinal cord by reconstruction of the intercostal artery (IA) as far as possible between the levels of T8 and L1. The technique for reconstruction of the IA has previously been described in detail [13]. Typically, the patent segmental arteries at the level of T8 to L2 were tailored as a patch and sutured to one branch of the four-branched graft. Cold or warm perfusion of renal vessels was not routinely utilized during our center. Except for emergency repair or parts of extent IV repair, cerebrospinal fluid drainage (CSFD) was routinely placed before anesthesia. In common, for patients without postoperative spinal cord deficits, CSFD was used to keep the intracranial pressure under 10 mmHg and was removed 72 h after the operation. For the patients who suffered from spinal cord deficits, CSFD was kept no more than 2 weeks; The intracranial pressure was adjusted under 10 mmHg and not less than 6 mmHg.

### Definitions

Operative mortality was defined as in-hospital deaths or patients who died within 30 d after discharge. Complications were considered permanent if they were present at the time of hospital discharge or death. Stroke was diagnosed based on the presence of neurological deficits and acute lesions observed on brain Computed Tomography.

Regarding adverse events, a composite endpoint was defined as operative death, paraplegia, acute renal failure or stroke. A pulmonary complication was defined as a respiratory failure, tracheotomy, a ventilation time greater than 48 h, or pulmonary infection. Unprotected ischemic time represented the total ischemic time without distal perfusion using a bypass. Reoperation means aortic-relevant intervention that was necessitated by repair failure or involved the extension of a contiguous repair.

### Patient follow-up

All survivors were followed up regularly by once a year through clinic visits, WeChat, and telephone calls. Enhanced Computed Tomography was recommended for the patient at 3 to 6 months after surgery. The methods to confirm clinical events included reviewing medical records of outpatient follow-up, analysis of post-discharge imaging data, examination of death records, contacting patients, relatives, and referring physicians for details on complications, reintervention, and cause of late death.

### Statistical analysis

Continuous variables are described as mean ± SD or median (interquartile range) and categorical variables as a number (percentage). The data between the two groups were compared using either the Mann–Whitney U-test for continuous variables or Student’s t-test or Fisher’s exact test for categorical variables. The normality of continuous variables was assessed by the Kolmogorov–Smirnov test.

Risk factors for operative mortality were identified with univariable and multivariable logistic regression model analysis. Preoperative or intraoperative factors whose univariate association with outcomes had a *p*-value of < 0.05 were entered into multivariable logistic regression models to identify independent predictors. Variables considered in the operative mortality models included explantation of the endograft, Crawford’s extent II repair, and emergency repair. Survival was estimated using the Kaplan–Meier method. All statistical analyses were performed using the SPSS 25.0 (IBM SPSS, Armonk, NY) and a two-sided *p*-value of < 0.05 was regarded as statistically significant.

## Results

### Baseline characteristics

From November 2016 to June 2021, we performed open TAAA repair on 65 patients who underwent previous TEVAR in Deltahealth Hospital Shanghai. Two patients with endograft infection were excluded from this study, leaving 63 patients for analysis. Of 63 consecutive patients who underwent TAAAR after TEVAR, 43 (68.3%) patients were male. MFS was present in 39 (61.9%) patients. The initial indications for TEVAR are listed in Table 1. The median interval time between TEVAR and TAAAR was 2.6 (interquartile range, IQR, 1.2–6.1) years. The mean age at the time of TAAAR was 41 ± 12 years. The extent of TAAA was extent I in 9 (14.3%), II in 22 (34.9%), III in 23 (36.5%), and IV in 9 (14.3%).

**Table 1:** Baseline characteristics

Variable	Whole (*n* = 63)
**Baseline characteristics**	
Age, year	41 ± 12
Male gender	43 (68.3)
Body mass index > 25	21 (33.3)
**Comorbidities**	
Hypertension	32 (50.8)
Smoking	21 (33.3)
Coronary artery disease	0 (0)
Prior stroke	0 (0)
Marfan syndrome	39 (61.9)
Prior re-TEVAR	26 (41.3)
**Indications for prior TEVAR**	
Residue type I dissection	17 (27.0)
Type III dissection	42 (66.7)
Aortic ulcer	1 (1.6)
Aortic rupture	3 (4.8)
**Prior proximal aortic surgery**	
Bentall operation	24 (38.1)
Ascending aortic replacement	6 (9.5)
Prior total arch replacement + frozen elephant trunk	11 (17.5)
**Endoleak**	22 (34.9)
Type I endoleak	18 (28.6)
I a	6 (9.5)
I b	6 (9.5)
I a + I b	6 (9.5)
Type II endoleak	4 (6.3)
Persisted false lumen perfusion	36 (57.1)
Aortic rupture	9 (14.3)
**Pathology of aneurysms**	
Degenerative	3 (4.8)
Post-dissection	60 (95.2)
The interval between TEVAR and TAAAR, year	2.6 (1.2–6.1)
Maximum distal aortic size, mm	71 ± 22
Positive intercostal artery angiogram	33 (52.4)
**Extent of TAAA**	
I	9 (14.3)
II	22 (34.9)
III	23 (36.5)
IV	9 (14.3)

Values are presented as n (%), mean ± SD or median (interquartile range)

*TAAAR* thoracoabdominal aortic aneurysm repair, *TEVAR* thoracic endovascular repair

### Operative data

Emergency TAAAR was applied in 10 (15.9%) patients, including 1 symptomatic aneurysm patient and 9 patients for containing aortic rupture. Stent grafts were removed in 31 (49.2%) patients (Table 2).

**Table 2:** Operative details

Variable	Whole (*n* = 63)
Emergency repair	10 (15.9)
Operation time, min	429 ± 130
**Distal perfusion strategy**	
Simple aortic clamping	8 (12.7)
Aorto-iliac bypass	20 (31.7)
Femoro-femoral bypass	35 (55.6)
Cardiopulmonary bypass	35 (55.6)
**Deep hypothermic circulatory arrest**	22 (34.6)
Circulatory arrest time, min	24 ± 8
Graft explantation	31 (49.2)
Visceral branch reattachment	50 (79.4)
***Clamp and ischemic times*, min**	
Proximal aorta	16 (0–21)
Intercostal artery, unprotected^a^	37 (23–58)
Superior mesenteric artery, unprotected^a^	33 (25–43)
Celiac artery, unprotected^a^	40 (30–57)
Maximal renal artery, unprotected^a^	44 (35–53)
Intercostal/lumbar artery reattachment	50 (79.4)
Reconstruction of left subclavian artery	14 (22.2)
Reconstruction of right iliac artery	37(58.7)
Cerebrospinal fluid drainage	44 (69.8)
Urine volume, ml	550 (360–750)
Estimated blood loss, ml	3000 (1200–4000)

Values are presented as n (%) or median (interquartile range). unprotected^a^ means that total time without perfusion during the operation

For 14 (22.2%) patients, additional reconstruction of the left subclavian artery was performed because of subclavian steal syndrome or subclavian artery occlusion before their TAAAR. One patient underwent removal of the spleen for a severe tear during the procedure. The intercostal or lumber artery was reattached in 50 (79.4%) patients, and CSFD was placed in 44 (69.8%).

### Early outcomes

#### Operative mortality and morbidity

Operative mortality was 17.5% (*n* = 11) due to stroke (*n* = 5), respiratory failure (*n* = 2), bowel necrosis (*n* = 1), low cardiac output (*n* = 1), and coagulopathy (*n* = 2) (Table 3). The details of operative mortality in subgroups are listed in Table 4. In the multivariable logistic regression models of operative mortality, explantation of the endograft was associated with increased risks (odds ratio, 6.14; 95% confidence interval [CI], 1.20–31.26; *p* = 0.029) (Table 5).

**Table 3:** Operative outcomes

Variable	Whole (*n* = 63)
Adverse events	18 (28.6)
Operative death	11 (17.5)
**Complications**	
Paraparesis	3 (4.8)
Paraplegia	7 (11.1)
Acute renal failure	6 (9.5)
Cerebral complications	7 (11.1)
Transient ischemia attack	2 (3.2)
Stroke	5 (7.9)
Pulmonary complications	19 (30.2)
Intubation time > 48 h	17 (27.0)
Redo tracheal intubation	10 (15.9)
Tracheostomy	8 (12.7)
Gastrointestinal dysfunction	4 (6.3)
Reoperation for bleeding	8 (12.7)
Contained bleeding	7 (11.1)
Renal artery occlusion	1 (1.6)
Extracorporeal membrane oxygenation	2 (3.2)
Continuous renal replacement therapy	7 (11.1)
Duration of hospital, d	25 (20–35)
ICU of length stay, d	3.7 (1.7–5.6)
Packed red blood cells, IU	16 (6–34)
Fresh frozen plasma, IU	22 (10–50)
Platelets, IU	1 (1–2)

Values are presented as n (%) or median (interquartile range)

*ICU* intensive care unit

**Table 4:** The details of operative mortality in subgroups

**Variable**	n, %
Male gender (*n* = 43)	8 (12.7)
Prior smoke (*n* = 21)	3 (14.3)
Hypertension (*n* = 32)	7 (21.9)
Body mass index > 25 (*n* = 21)	3 (14.3)
Prior re-TEVAR (*n* = 26)	7 (26.9)
Prior frozen elephant trunk (*n* = 11)	3 (27.3)
**Endoleak (*n* = 22)**	6 (27.3)
Type I endoleak (*n* = 18)	4 (22.2)
I a (*n* = 6)	1 (16.7)
I b (*n* = 6)	2 (33.3)
I a + I b (*n* = 6)	1 (16.7)
Type II endoleak (*n* = 4)	2 (50.0)
Mafan syndrome (*n* = 39)	5 (12.8)
**Extent of TAAA**	
I (*n* = 9)	1 (11.1)
II (*n* = 22)	7 (31.8)
III (*n* = 23)	2 (8.7)
IV (*n* = 9)	1 (11.1)
Persisted false lumen perfusion (*n* = 36)	8 (22.2)
**Pathology of aneurysms**	
Degenerative (*n* = 3)	0 (0)
Post-dissection (*n* = 60)	11 (18.3)
Emergency procedure (*n* = 10)	3 (30.0)
Aortic rupture (*n* = 9)	3 (33.3)
Simple aortic clamping (*n* = 8)	1 (12.5)
Aorto-iliac bypass (*n* = 20)	3 (15.0)
Femoro-femoral bypass (*n* = 35)	7 (20.0)
Deep hypothermic circulatory arrest (*n* = 22)	6 (27.3)
Cardiopulmonary bypass (*n* = 35)	7 (20.0)
Visceral branch reattachment (*n* = 50)	10 (20.0)
Reconstruction of the right iliac artery (*n* = 37)	5 (13.5)
Explantation of endograft (*n* = 31)	9 (29.0)
Without explantation of endograft (*n* = 32)	2 (6.3)

Values are presented as number of deaths in this subgroup/total number in this subgroup (%)

*TAAA* thoracoabdominal aortic aneurysm, *TEVAR* thoracic endovascular repair

**Table 5:** Analysis of risk factors for operative mortality

Variable	Univariable		Multivariable	
	**OR (95% CI)**	***P* value**	**OR (95% CI)**	***P* value**
Explantation of endograft	6.136 (1.205–31.255)	.029	6.136 (1.205–31.255)	.029
Crawford’s extent II repair	4.317 (1.1–16.939)	.036		
Emergency repair	2.411 (0.513–11.33)	.265		
Marfan syndrom	0.441 (0.118–1.647)	.233		
DHCA	2.70 (0.718–10.157)	.142		
Femoro-femoral bypass	1.50 (0.391–5.752)	.554		
Aorto-iliac bypass	0.772 (0.181–3.284)	.726		
Simple aortic clamping	0.643 (0.071–5.828)	.643		

*OR* odds ratio, *CI* confidence interval, *DHCA* deep hypothermic circulatory arrest

Paraplegia occurred in 7 (11.1%) patients, and paraparesis occurred in 3 (4.8%). Six (9.5%) patients developed acute renal failure needing temporary dialysis; Of these, 3 died during hospitalization, and 3 patients recovered before hospital discharge. The operative death and the major complications of surgical techniques are listed in the Supplementary Table 1.

### Midterm outcomes

Clinical follow-up was fully completed at a mean 3.3 ± 2.0 (range 0.1–6.7) years. CT images were obtained from all surviving patients at a median 1.2 years (interquartile range: 0.1–2.8). Intercostal artery (IA) dilation after TAAAR occurred in 6 patients at median of 1.0 years (range 0.1–2.5), which included IA dilation in 6 that was exclusively seen in MFS (17.6% [6/34] vs. 0, *p* = 0.081). The IA occlusion was found in three patients (0.03 years, 0.26 years and 0.92 years after TAAAR, respectively). Significant left renal artery stenosis occurred in 1 patient 0.8 years after surgery.

As confirmed by PET-CT, new onset of aortic graft infection occurred in 3 patients. One patient suffered from graft infection below the level of the celiac trunk after receiving extent II repair at 0.4 years and was alive at the latest follow-up. One patient suffered from mediastinal infection after receiving extent III repair at 2.8 years. He then underwent bilateral pectoralis major muscle advancement flaps and survived. One patient suffered from graft infection from the level of the aortic arch from the level of the iliac artery after receiving extent III repair at 3.7 years. He then died after 2 months.

#### Survival and reoperation

**Fig. 1:**
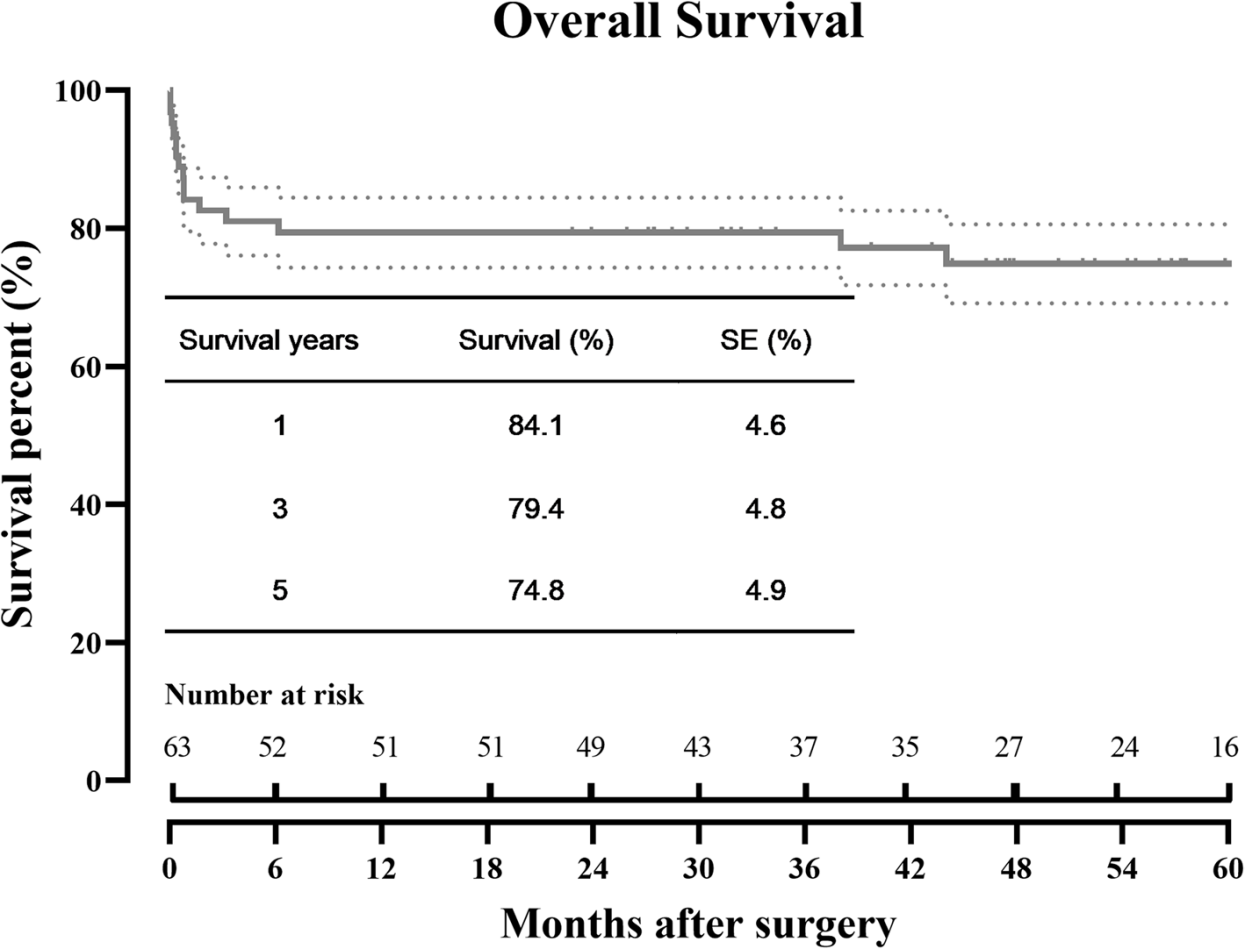
Overall survival of open thoracoabdominal aortic aneurysm repair after thoracic endovascular aortic repair

Four patients died during our follow-up. Of these, 2 patients died at 4 months due to aorto-tracheal fistula after TAAAR. Two patients died at 3 years due to sepsis and intracerebral hemorrhage, respectively. At 5 years, the estimated survival was 74.8 ± 4.9% (Fig. 1).

Late reoperations were done in 2 patients due to developing acute DeBakey II type dissection at 2.5 years and 1.3 years, respectively. One patient was treated by a replacement of the ascending aorta and total aortic arch combined with frozen elephant trunk implantation. One patient received Bentall and frozen elephant trunk with total arch replacement.

## Discussion

In line with previous reports [8], the results of this presented study show that TAAAR after prior TEVAR is associated with a higher risk of morbidity and mortality compared with the standard open TAAAR. However, the mid-term outcomes in this study showed that TAAAR after TEVAR was durable. Open TAAAR after prior TEVAR should serve as a feasible salvage treatment if there is no other therapeutic option (conservative treatment or endovascular therapy) when performed at a high-volume center.

Open repairs of TAAA diseases, secondary to complications of TEVAR, tended to face great risks. The operative mortality (17.5%) in this study was higher compared with other studies of open TAAAR from European and American experienced centers reporting their operative mortality was 7.5%–8.9% [9, 14, 15]. The risk factors on operative mortality in this study showed that TAAAR requiring the explantation of endograft was prone to result in significantly higher mortality (29.0% vs. 6.3%; *p* = 0.022) (Table 4). Furthermore, the multivariable model of operative mortality demonstrated that explantation of endograft was at higher risk of operative death (OR, 6.14; 95% CI, 1.20–31.26; *p* = 0.029) (Table 5). We consider that maybe partly attributed to that explantation of endograft may cause damage to the intima of the aorta. Dropping thrombus during this procedure may give rise to extensive embolism, particularly in the cerebrovascular system, which may be a reasonable explanation for the high incidence of cerebral complications (11.1%) exclusively occurring in patients with an extraction of the endograft in this study. Furthermore, explantation of the endograft was associated with more extensive disease and complexity and may be a surrogate for the severity of the disease.

Other factors also contributed to the complexity and difficulty of TAAAR after TEVAR. First, the complications of TEVAR (endoleak, graft misplacement) may accelerate the enlargement of thoracic aortic aneurysm, which could cause compression, adhesion, and even fistula between the descending aorta and peri-aortic organs; Second, a peri-aortic inflammatory response caused by the endograft might lead to extensive adhesion between the aorta and lung. Thus, severe complications, such as bleeding and respiratory insufficiency, could occur during surgical exposure and dissecting. Finally, the limited use of the reconstruction of the intercostal artery due to occlusion of the targeted artery after TEVAR could increase the risk of spinal cord ischemia.

The demographic characteristics of this cohort were noteworthy. The mean age of patients at their TAAAR (41) was relatively young, with a low prevalence of comorbidities. In addition, MFS was present in 61.9% of patients who underwent their initial TEVAR in the local cardiac center. With the development of materials, experience, and stent grafting, due to its superiority of less invasiveness and favorable short-term outcomes, the application of endovascular repair is growing extensively not only in aneurysm and trauma but also in acute and chronic aortic dissection [16–18]. However, the need for reintervention is not rare, and the requirement for open repair has been on the increase according to the previous reports [19, 20]. Shalhub and colleagues reported that TEVAR has been applied in young patients with heritable connective tissue disorder [21]. Unfortunately, the incidence of reinterventions was up to 41.9% at a median of 2 years during follow-up. Considering the high incidence of revision and risk of secondary open repairs, operators should weigh against the long-life expectancy and the risk of aneurysm rupture when adopting the strategy of endovascular repair, particularly in young patients or patients with MFS.

The midterm prognosis in this study seems respectable. However, patients who undergo TAAAR after TEVAR may remain at risk of future aortic events such as infection of implantations and massive hemoptysis caused due to aorto-esophageal fistula. In this study, late intercostal artery dilation occurred in 6 patients with MFS, and 2 patients who did not have bronchial-related diseases in the past died of massive hemoptysis within 4 months after their hospital discharge. Thus, it is critical to recommend patients carry out lifelong postoperative imaging surveillance for early and timely intervention.

Similar to previous reports [9, 22], pulmonary complications remained the most frequent complication. Although several measures in this study were used to improve the pulmonary function pre-operatively if possible, in the elective cases, the pulmonary complications may not be evitable if patients require urgent operation with huge thoracic aortic aneurysm. On the one hand, left lung atrophy could occur due to persistent compression by the progressing expansion of the descending aortic aneurysm. On the other hand, extensive trauma while dissecting for surgical exposure might not be avoided because of severe adhesion between the lung and the descending aorta.

## Study limitations

Limitations of our study include its single center and retrospective design. Although the treatment approach was determined by multidisciplinary discussion, selection bias cannot be avoided. Moreover, most TEVARs were performed at an outside institution. Thus, we could not evaluate the true incidence of complications of TEVAR that require open TAAAR. In addition, the number of patients was insufficient, and the period of follow-up was not long enough. Due to many missing data in long-term imaging, it was not included in the analysis of this study. Consequently, it was difficult to provide stable evidence on the outcome of TAAAR after TEVAR both in perioperative and long-term results. Finally, the feature of TAAAR secondary to TEVAR with substantial mortality and morbidity means that it could not be generalizable except to experienced centers.

## Conclusions

In this series of TAAA after TEVAR, open repair was related with a high risk of operative mortality and morbidity. The follow-up outcomes were encouraging and durable in terms of late death and reoperation. Open TAAAR after prior TEVAR should serve as a feasible salvage treatment even in the endovascular era and should be limited to dedicated centers due to the significant operative mortality and morbidity.

### Supplementary Information

**Additional file 1: Supplementary Table 1. **The operative death and the major complications of surgical techniques.

## Data Availability

The datasets used during the current study are available from the corresponding author on reasonable request.
