# Supplementary material for: Early and mid-term outcomes of open thoracoabdominal aortic aneurysm repair after thoracic endovascular aortic repair
Source: BMC Cardiovasc Disord. 2024 Mar 26;24:182. doi: 10.1186/s12872-024-03837-8 (PMC10964665; doi:10.1186/s12872-024-03837-8)
Supplement: Supplementary file 1 — Additional file 1: Supplementary Table 1. The operative death and the major complications of surgical techniques. [file 12872_2024_3837_MOESM1_ESM.docx]

**Supplementary table 1.** The operative death and the major complications of surgical techniques

| **Surgical techniques** | Operative death | Stroke | Paraplegia | ARF |
| --- | --- | --- | --- | --- |
| Simple aortic clamping (n = 8) | 1 (12.5) | 0 (0) | 0 (0) | 0 (0) |
| Aorto-iliac bypass (n = 20) | 3 (15.0) | 1 (5.0) | 2 (10.0) | 1 (5.0) |
| Femoro-femoral bypass (n = 35) | 7 (20.0) | 4 (11.4) | 5 (14.3) | 5 (14.3) |
| DHCA (n = 22) | 6 (27.3) | 3 (13.6) | 4 (18.2) | 4 (18.2) |

Values are presented as n (%); DHCA, deep hypothermic circulatory arrest; ARF, acute renal failure.
